# Bisphosphonates in Langerhans Cell Histiocytosis: An International Retrospective Case Series

**DOI:** 10.4084/MJHID.2016.033

**Published:** 2016-07-01

**Authors:** Deepak Chellapandian, Polyzois Makras, Gregory Kaltsas, Cor van den Bos, Lamia Naccache, Raajit Rampal, Anne-Sophie Carret, Sheila Weitzman, R. Maarten Egeler, Oussama Abla

**Affiliations:** 1 Division of Hematology/Oncology and Bone Marrow Transplantation, Department of Pediatrics, The Hospital for Sick Children (SickKids), University of Toronto, Toronto, Ontario, Canada; 2 Department of Endocrinology & Diabetes, 251 Hellenic Airforce & VA General Hospital, Athens, Greece; 3 Department of Pathophysiology, National and Kapodistrian University of Athens, Athens, Greece; 4 Department of Pediatric Oncology, Emma Children’s Hospital/Academic Medical Center, Amsterdam, The Netherlands; 5 Hémato-oncologue pédiatrique, Centre hospitalier universitaire de Québec, Quebec City, Canada; 6 Leukemia Service, Memorial Sloan-Kettering Cancer Center, New York, USA; 7 Division of Hematology-Oncology, Department of Pediatrics, Centre Hospitalier Universitaire (CHU) Sainte-Justine, Université de Montréal, Montreal, Quebec, Canada

## Abstract

**Background:**

Bone is the most common organ of involvement in patients with Langerhans cell histiocytosis (LCH), which is often painful and associated with significant morbidity from pathological fractures. Current first-line treatments include chemotherapy and steroids that are effective but often associated with adverse effects, whereas the disease may reactivate despite an initial response to first-line agents. Bisphosphonates are osteoclast inhibitors that have shown to be helpful in treating bone lesions of LCH. To date, there are no large international studies to describe their role in treating bone lesions of LCH.

**Method:**

We conducted a multicenter retrospective review of 13 patients with histologically proven LCH, who had received bisphosphonates either at diagnosis or at disease reactivation.

**Results:**

Ten patients (77%) had a single system bone disease, and 3 (23%) had bone lesions as part of multisystem disease. Median follow-up time post-bisphosphonate therapy was 4.6 years (range, 0.8 to 8.2 years). Treatment with bisphosphonates was associated with significant pain relief in almost all patients. Twelve (92%) achieved resolution of active bone lesions, and 10 out of them had no active disease for a median of 3.5 years (range, 0.8 to 5 years). One patient did not respond. No major adverse effects were reported in this series.

**Conclusion:**

Bisphosphonates are well-tolerated drugs that can significantly improve bone pain and induce remission in active bone LCH. Future prospective studies evaluating the role of bisphosphonates in LCH are warranted.

## Introduction

Langerhans cell histiocytosis (LCH) is a dendritic cell (DC) neoplasm defined by the presence of pathologic cells with Langerhans cell features that are positive for CD1a, Langerin (CD207) and S100 protein.^1^ The disease varies widely in clinical presentation from localized involvement of a single bone to a fatal disseminated life-threatening disease involving risk organs such as liver, spleen, or hematopoietic system.^2^ Bone is the commonest area of involvement in about 80% of patients, which can be painful and associated with significant morbidity from pathologic fractures. Treatment for bony lesions includes surgical curettage, intralesional infiltration of corticosteroids,^3^ low-dose irradiation, indomethacin or systemic chemotherapy.^4^

Bisphosphonates are chemical analogues of pyrophosphates that act by inhibiting osteoclasts, thus preventing bone resorption.^5^ They were initially found to be effective in multifocal eosinophilic granuloma of bone (former description of LCH bone lesions) in 1989.^6^ Subsequently, this beneficial effect of bisphosphonates in bone lesions of LCH was confirmed in several other case reports.^7^–^10^ In 2005, da Costa et al showed that multi-nucleated giant cells (MGCs) in LCH express several osteoclast markers and responsible for producing osteoclast-inducing cytokines.^11^ These osteolytic cytokines along with various other matrix-degrading enzymes produced by the MGCs are involved in inducing osteolysis; thus, a rationale for using bisphosphonates in LCH was established.

Currently, chemotherapy and steroids remain the standard treatment in most patients with LCH-related bone disease. Due to immediate and long-term adverse effects related to chemotherapy and problems of recurrent reactivations despite standard treatment, less toxic options like bisphosphonates become attractive. This report summarizes the international experience describing the role and safety of using bisphosphonates for patients with bone involvement by LCH.

## Material and Methods

### Data collection

Survey documents were developed at the Hospital for Sick Children (Toronto), and distributed to five LCH treating centers across North America and Europe. The participating centers included Toronto and Quebec City (Canada), New York City (USA), Athens (Greece), and Amsterdam (The Netherlands). Appropriate ethics approval was obtained from all participating centers. Data were collected on patients diagnosed with LCH and treated with bisphosphonates as single agents between 1995 to 2014, and included age at diagnosis, gender, sites of disease, disease status (initial diagnosis and number of reactivations), initial treatment received, type and dose of bisphosphonates used, number of courses, response to bisphosphonate therapy including pain grades and radiological assessment pre- and post-therapy, toxicity, and long-term outcome.

### Data analysis

Statistical analyses were completed using SAS 9.4 (SAS Institute Inc., Cary, NC, USA). Categorical variable were summarized using counts and percentages. Descriptive statistics summarized continuous variables.

### Evaluation criteria and definitions

The National Cancer Institute’s Common Terminology Criteria for Adverse Events (CTCAE)^12^ was used to define the pain response pre- and post-bisphosphonate therapy; 0 for no pain, 1 for mild pain, 2 for moderate pain, and 3 for severe pain. The “best response” in bone lesions following bisphosphonates was evaluated using the disease state categories proposed by LCH-III protocol.^13^ The “no active disease” (NAD) status was defined as the disappearance of all signs and symptoms of disease with the exception of diabetes insipidus (DI) and central nervous system degeneration (CNS-ND), or residual radiological findings of bone lesions showing regression or stabilization with bone remodeling. The “no response” (NR) status was defined as unequivocal enlargement of the size of the existing bone lesion and/or appearance of new lesions. In this study, we acknowledged the variability in the response of bone lesions in children and adults, and exercised caution while interpreting the post-therapy response individually. An experienced radiologist at the respective institution assessed and evaluated the radiological findings.

## Results

The characteristics and treatment outcome of all the patients are summarized in Table 1. Thirteen patients (male/female ratio, 8:5 and age range, 2.8 to 55 years) with histologically proven LCH were included in this series. Ten patients (77%) had a single system bone disease, and 3 (23%) had bone lesions as part of multisystem disease. None of the 3 patients with the multisystem disease had risk organ (liver, spleen and/or hematopoeitic system) involvement. The median age at initiation of bisphosphonate therapy was 21.4 years (range, 2.7 to 55.3 years) and the median post- bisphosphonate therapy follow-up period was 4.6 years (range, 0.8 to 8.2 years).

### Management of LCH with bisphosphonates

Four patients (31%) received pamidronate, 3 (23%) received alendronate, and 6 (46%) received zoledronate. All children in the series received either pamidronate or alendronate therapy while all adults received zoledronate. Pamidronate was administered intravenously (IV) at a median dose of 1 mg/kg/course between 3 to 6 “monthly” courses and alendronate was administered orally as a single “daily” dose of 5 mg or “weekly” course of 70 mg. Most of the patients who were treated with zoledronate received a single IV dose of 5 mg, while one patient received a “monthly” course of 4 mg. Most of the patients had received some form of chemotherapy and/or radiotherapy and had progressed before starting bisphosphonate therapy, while a few received bisphosphonates as upfront therapy. No other LCH-directed treatment was administered during the course of bisphosphonate therapy except for desmopressin (DDAVP) in patients with DI (nos. 3, 9) and hydrocortisone replacement therapy for pituitary insufficiency in one patient (no. 4). Patient 10 received a short course of steroids and yet continued to have active bone disease prior to starting therapy with bisphosphonates.

### Clinical effects of bisphosphonates

Twelve of 13 patients (92%) achieved NAD with radiological re-ossification and normalization of active bone lesions either during or after cessation of bisphosphonates. Of the 12 patients who had obtained NAD, 10 continued to have a complete radiographic resolution for a median of 3.5 years (range, 0.8 to 5 years) since the commencement of bisphosphonates. One patient (no. 5) had bone reactivation 8 months following cessation of bisphosphonates and subsequently achieved NAD with methotrexate therapy. One child (no. 11) who received pamidronate for his third bone reactivation did not respond and continued to progress. The same patient achieved NAD after treatment with multiple chemotherapeutic agents. Patient no. 3 developed radiographic central nervous system neurodegeneration (CNS-ND) 2 years after stopping pamidronate therapy. However the radiographic findings remained stable during the last follow up without further therapeutic intervention.

Eleven of 13 patients reported either moderate or severe pain prior to starting therapy with bisphosphonates and required analgesic medications. Seventy five percent (75%) of them reported no pain during or after cessation of bisphosphonate therapy along with restoration of functional status, while the rest reported improvement to only mild pain.

**Figure 1:**
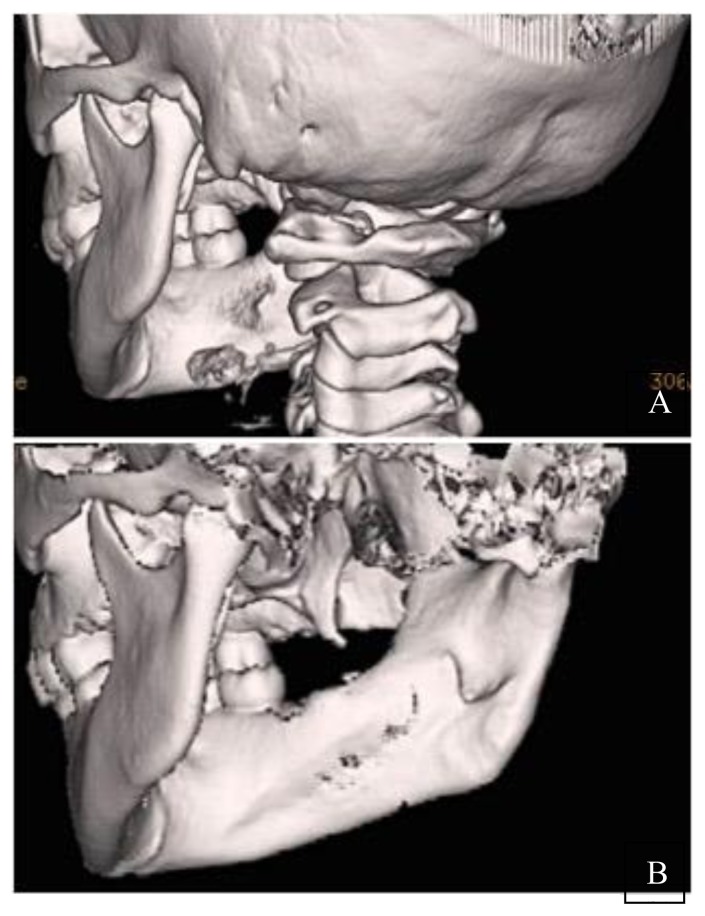
3-dimensional CT of mandible before (a) and after (b) 3 courses of pamidronate therapy. a) Osteolytic lesions in the ascending ramus, neck and condylar head of right mandible. b) Bone remodeling with thick periosteal reaction.

**Figure 2:**
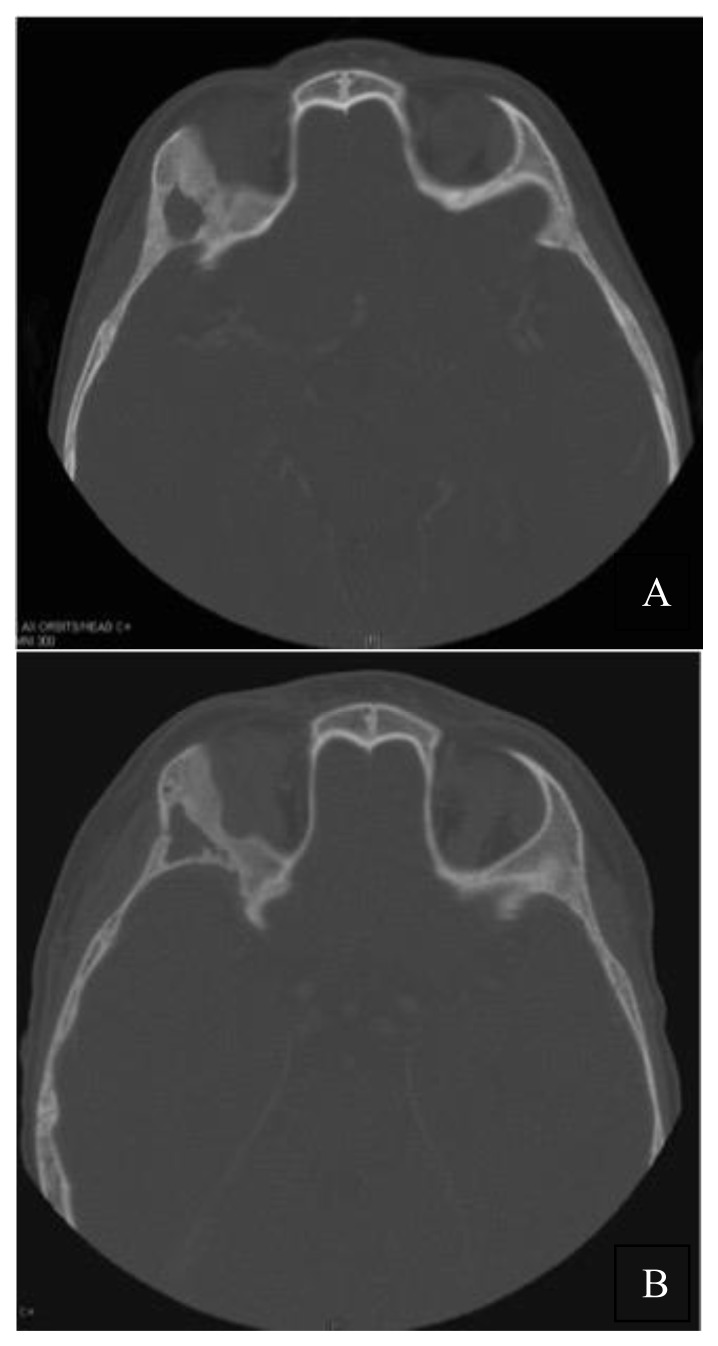
Skull CT before (a) and after (b) 2 courses of pamidronate therapy. a) Permeative bone lesions in the lateral wall of orbit and soft tissue mass involving the right lacrimal gland and lateral rectus muscle. b) Bone remodeling and reduction in orbital soft tissue mass

In Figure 1, a complete resolution of mandibular lytic lesions following pamidronate therapy is demonstrated in a 3-dimensional CT scan. In Figure 2, CT scan showed bone remodeling and reduction in orbital soft tissue mass following pamidronate therapy.

### Adverse effects

Bisphosphonate therapy was well tolerated by all patients without major adverse effects. One patient (no. 11) had a fever during initial courses of IV pamidronate administration and another (no. 10) had mild elevation in parathyroid hormone (PTH) levels following alendronate.

## Discussion

In the present study, bisphosphonates appear to be an effective option in treating bone lesions of LCH. The majority of patients in this series demonstrated significant improvement in pain symptoms related to the disease with return of functional status and 92 % were able to achieve complete resolution of active bone lesions. Several case reports have been published suggesting the beneficial effects of bisphosphonates in LCH, but there is currently no international consensus on the role of bisphosphonates in LCH. A nationwide survey from Japan comprising of 16 children with LCH investigated the role of pamidronate in reactivated LCH and concluded that pamidronate was effective in the resolution of bone lesions in 75% of children with acceptable toxicity profile.^14^ Our report is the first international study that included both children and adults, and described the role and safety of various bisphosphonates in the treatment of bone lesions of LCH suggesting that this is a class effect.

Bisphosphonates have been used as standard of care for bone pain caused by various metastatic tumors,^15^,^16^ and interestingly, the bone pain of LCH shares similar properties with cancer-induced pain. LCH cells, like tumor cells, release certain cytokines including Tumor Necrosis Factor (TNF)- α and Interleukin-1^17^,^18^ that stimulate osteoclast activity leading to fragility of bone and increased fracture risk. Moreover, both receptor activator of nuclear factor B ligand (RANKL) and osteoprotegerin (OPG), which are crucial key factors for the maturation and activation of osteoclasts, have recently been implicated in the disease process both at the lesional and systemic level.^19^–^21^ Amelioration of pain symptoms combined with the resolution of bone lesions is probably explained by the anti-osteoclastic property of bisphosphonates that helps reduce noxious inflammatory substances and other matrix degrading cytokines in the active lesions, thereby conferring its analgesic and bone-remodeling properties.

Pamidronate is the only bisphosphonate that is most widely used in children for various bone conditions with a well-established safety profile.^22^,^23^ Similarly, the safety and efficacy of zoledronate has been extensively reviewed in adults, and especially found to be effective in decreasing the risk of skeletal-related events secondary to breast cancer such as pathological fractures, spinal cord compressions and hypercalcemia.^24^ Although there are few reports demonstrating the role and safety of alendronate in certain skeletal conditions such as avascular necrosis and osteoporosis, large-scale prospective studies evaluating its safety profile and efficacy is still lacking.^25^ Despite the small sample size of this series, we were able to observe clinical activity with a wide range of bisphosphonates at different doses. However no positive correlation could be established between the type of agent used, the dose administered and outcomes.

Bisphosphonates especially pamidronate has shown some efficacy for nonostotic LCH such as skin and soft tissue lesions.^14^ This concurs with the laboratory findings of da Costa et al who demonstrated the presence of CD68+ osteoclast-like MGCs in nonostotic lesions that also co-express CD1a.^11^ The assessment of response in nonostotic lesions was not the intent of this study, but interestingly one child (no. 2) in our series demonstrated significant response in skin and soft tissue lesions following pamidronate therapy (Figure 2). Given the varying natural course of the disease, no favorable conclusion can be derived from this favorable response; however the effect of bisphosphonates in nonostotic lesions cannot be completely ruled out and need to be further explored in a large prospective study.

The most commonly reported toxicities with bisphosphonates are the acute phase reaction and hypocalcemia,^16^ the latter especially among vitamin D deficient patients. One patient in our series developed mild fever with initial courses of bisphosphonate that responded to antipyretics. PTH abnormality was reported in one patient manifesting as secondary hyperparathyroidism due to mild hypocalcemia, which was appropriately monitored and managed. It is possible that the PTH abnormality following bisphosphonates could be underreported in this series, as not all the patients would have been systematically tested for it during the therapy. Another rare yet significant side effect associated with IV bisphosphonates is osteonecrosis of the jaw (ONJ);^26^ however none of the patients in our cohort was reported with ONJ. In addition, ONJ is almost never seen in children who only receive a much lower dose of bisphosphonates to treat bone lesions.^27^ Thus, both oral and intravenous bisphosphonates appear to be a safe option to treat bone lesions of LCH in adults as well as in children. Ensuring adequate vitamin D repletion prior to bisphosphonate therapy and monitoring serum calcium and PTH pre- and during bisphosphonate therapy is highly recommended.

Our study has a few limitations that warrant consideration. The retrospective nature of the study can potentially introduce reporting biases and confounding factors, which makes interpretation of these data challenging. The longer study period, approximately 19 years, precluded us from performing a central radiographic review. Nevertheless, our data suggests that bisphosphonates might be an effective treatment option for symptomatic pain relief and resolution of active bone lesions in patients with LCH, and may obviate the need for toxic chemotherapy in less advanced cases. Future prospective studies are required to optimize the strategy of bisphosphonate treatment in LCH, including the ideal agent in different age groups, the timing of treatment initiation, optimal dose and duration of therapy, and long-term efficacy and safety.

## Figures and Tables

**Table 1:** Characteristics and Outcome of Patients with LCH Who Received Bisphosphonate Therapy.

Pt #	At the start of BP therapy			BP		Response after BP therapy		Toxicity	Reactivation after maximum response	Subsequent therapy	Survival post-BP therapy
	Age/Sex	Disease status	Lesions	Type	Dose	Best response	Description				
1	5y 8m/M	3rd Re	Rt. mandible, C5 vertebra	Pam (IV)	1mg/kg/day × 3 days q monthly	NAD after 6 courses	Remodeling and thick periosteal reaction of mandibular lesion, stable C5 lesion	None	None	None	NAD at 4.2y+
2	2y 8m/M	1st Re	Skull, facial bones, scalp, skin	Pam (IV)	1mg/kg/day × 3 days q monthly	NAD after 4 courses	Stabilization of skull and facial bone lesions, significant reduction of soft tissue and skin lesions	None	Rad-ND after 2 years	None	NAD at 8y+
3	36y/F	Diag	Rt. femur, multiple ribs, lung, DI	Alen (PO)	70 mg q weekly	NAD after 2 years	Sclerotic 1 cm lesion of rt. femur and minimal sclerotic lesions in 2 ribs	None	None	None	NAD at 3y+
4	27y/M	1st Re	Mandible, rt. hip, femur	Zol (IV)	5 mg once	NAD	Sclerotic intraosseous changes around the right hip osteolytic lesion, no change in mandible and femur	None	None	None	NAD at 10m+
5	21y/M	Diag	Skull, both femurs, left tibia	Zol (IV)	5 mg once	NAD	Sclerotic changes in the left femur and tibial osteolytic lesions, no change in skull lesions	None	Re in bone after 8 months	MTX	NAD at 1.7y+
6	20y/M	1st Re	Lumbar vertebra, both tibias	Zol (IV)	5 mg once	NAD	Sclerotic changes in the tibial osteolytic lesions, other bone lesions stable	None	None	None	NAD at 3.6y+
7	32y/F	1st Re	Skull	Zol (IV)	5 mg once	NAD	Stable skull lesions	None	None	None	NAD at 2y+
8	25y/F	1st Re	Skull, iliac bone	Zol (IV)	5 mg once	NAD	Reduction in size and sclerosis of osteolytic skull lesions, other bone lesions stable	None	None	None	NAD at 2y+
9	55y/F	Diag	Skull, mandible, both femurs, both tibias, ribs, iliac bone, DI	Alen (PO)	70 mg q weekly	NAD after 4 years	Significant reduction in skull lesions, other bone lesions stable	None	None	None	NAD at 1y+
10	9y 3m/F	3rd Re	C4 vertebra, corpus of L3 vertebra, iliac bones, rt. pubic ramus	Alen (PO)	5 mg daily	# NAD after 2.5 years	C4 lesion- non-evaluable (treated with bone graft). Complete resolution of lesions in L3 vertebra, iliac bones and pubic ramus.	Mild increase in PTH	None	None	NAD at 5y+
11	4y 2m/M	3rd Re	Skull, left scapula	Pam (IV)	1mg/kg × 3 days q monthly	NR after 3 courses	No response	Mild fever	Progression	2-CDA, MP, ARA-C, VCR, Indocin	NAD at 7.1y+
12	45y/M	Diag	T9 & T10 vertebra, ribs, sacrum, pelvis, ischial tuberosity, rt. acetabulum, femur	Zol (IV)	4 mg q monthly	NAD after 13 courses	Complete clearance of all lytic bone lesions except for mottled density in the pelvis and proximal femur	None	None	None	NAD at 3y+
13	6y 1m/M	2nd Re	Skull, left 6th rib, L2 vertebra	Pam (IV)	1mg/kg × 1 day q monthly	NAD after 6 courses	Complete resolution of all bone lesions	None	None	None	NAD at 1.1y+

Pt, patient; F, female; M, male; BP, bisphosphonates; Re, reactivation; Diag, at diagnosis; DI, diabetes insipidus; Rt, right; Pam, pamidronate; Alen, alendronate; Zol, zoledronate; IV, intravenous; PO, per os; DDAVP, desmopressin; HC, hydrocortisone; MTX, methotrexate; NR, no response; NAD, no active disease (apart from DI and CNS-ND); IV, intravenous; NR, no response; PTH, parathyroid hormone; Rad-ND, radiological neurodegeneration; 2-CDA, cladribine; MP, methylprednisone; ARA-C, cytarabine; VCR, vincristine; Indocin, Indomethacin.

# Pt received a short course of steroids and continued to have active disease in the bones prior to starting bisphosphonates.
